# The Associations Between Participation in Leisure Dance Activity, Perceived Health Status, Happiness Level, and Perceptions of Leisure Amidst Selected Demographic Determinants

**DOI:** 10.3390/healthcare14020144

**Published:** 2026-01-06

**Authors:** Seungok An, Wi-Young So, Jeonga Kwon

**Affiliations:** 1 Department of Special Physical Education, Yongin University, Yongin 17092, Republic of Korea; skyforve@yiu.ac.kr; 2 Department of Sports Medicine, College of Humanities, Korea National University of Transportation, Chungju-si 27469, Republic of Korea; 3 Department of Elementary Education, College of First, Korea National University of Education, Cheongju 28173, Republic of Korea

**Keywords:** happiness level, leisure dance activity, leisure perceptions, perceived health

## Abstract

**Objectives/Background**: In this study, we aimed to examine the associations of participation in leisure dance activity with perceived health status, happiness level, and perceptions of leisure, and the relevance of demographic characteristics to these associations. Moreover, we sought to explore ways of revitalizing leisure dance activity. **Methods**: We used data from the 2022 Korea National Leisure Activity Survey organized by the Korean Ministry of Culture, Sports, and Tourism. The 2022 Korea National Leisure Activity Survey was conducted from September to November 2022. The survey was conducted among 10,046 Koreans aged ≥15 who lived in 17 cities and provinces across the country. The participants were informed about the survey schedule in advance, and interviews were conducted at the participants’ homes using tablet PCs. Random telephone verification was performed on the recovered questionnaires to ensure the accuracy of responses. Questionnaires that passed the first verification were subjected to secondary verification by a computerized program, and questionnaires that did not pass the verification were supplemented and re-examined. The collected data were entered electronically through an encoding process, and only the data that passed the final test were compiled in the multi-stage verification process. The data were analyzed using frequency analysis, chi-squared tests, and multivariate logistic regression analysis. **Results**: Of the 1004 participants, 655 (6.5%) participated in leisure dance activity. Women, individuals in their twenties and thirties, college graduates or those with lower-level educational qualifications, and unmarried individuals were more likely to participate in leisure dance activities. In addition, leisure dance activities were found to be likely to increase happiness levels and leisure life satisfaction, and leisure expenses were likely to be low. **Conclusions**: Schools should provide high-quality dance education and enhance the professionalism of physical education teachers in the management of dance classes. This is because dance-related experiences in childhood can increase the likelihood of engaging in dancing in adulthood. Efforts are also needed to increase men’s awareness of and participation in leisure dance activities and to lower barriers to entry. The convergence of dance, games, and technology can make this possible.

## 1. Introduction

Work–life balance is a prominent topic in Korean society, with a broad social consensus recognizing its necessity [1]. In this context, leisure is seen as an essential aspect of life that is supportive of relaxation, in addition to self-fulfillment and social engagement. Engaging in leisure activities enables individuals to forge unique identities and participate in creative pursuits, fostering connections with others and promoting balance in their daily lives, thereby enhancing their quality of life. Moreover, Iwasaki [2] asserted that leisure offers avenues for coping with and recovering from stress, as well as facilitating personal growth and development. Therefore, leisure activities are recognized as an important entity in subjective well-being because they provide opportunities for people to attend to their values and needs [3].

Su et al. [4] broadly classified leisure activities as cognitive, physical, or social leisure activities. Cognitive leisure activities involve consciousness and intellect, such as watching television, reading newspapers, writing, drawing, and browsing the Internet. Physical leisure activities include body movements, such as dancing, gymnastics, running, walking, and swimming. Finally, social leisure activities encompass interactions with others, such as meeting friends, volunteering, and engaging in group-based religious activities [4]. Many people prefer physical leisure activities, as body movement improves cognitive ability and interpersonal relationships, thus providing the benefits of both cognitive and social leisure activities [5,6]. In particular, dance sports, such as ballet, yoga, Pilates, line dance, Tae-Bo, and jive, allow people to express their emotions through body movements [7]. Moreover, Lanke and Nath [8] stated that dancing, moving the body to rhythmic music, as a leisure activity can improve physical health and reduce psychological and social stress. In Korean society, dance is performed for leisure, socializing, and improving health. In the past, dance has not been considered a beneficial leisure activity but rather a source of entertainment and playfulness. However, Yu and Han [9] claimed that, in modern times, it has gradually been accepted as a beneficial leisure activity. Dancing has salient effects on one’s physical health [8,10,11,12,13]. Specifically, participation in leisure dance activities have a positive effect on physical health [8,10,11,12,13]. For example, Lanke and Nath [8] found that those who engaged in dancing reported significantly higher physical activity levels than those who did not engage in dancing. In addition, participation in leisure dance activity had a positive effect on improvements in cardiorespiratory fitness, body composition, and inflammatory biomarkers [11]; muscle strength and balance and lower limb strength and adherence rates [12]; and cardiorespiratory endurance, balance, lower limb strength and endurance, physical agility, and flexibility, and reduced body fat [13]. Participation in leisure dance activity should be highly encouraged because of its positive effects on physical health. However, people also suffer from physical injuries from dancing. For example, Smith et al. [14] suggested that in the process of ballet, musculoskeletal injuries were severe, and ballet participants experienced lumbosacral pain, painful snapping hip, patellofemoral pain, and foot and ankle pain [14]. The authors suggested that dancing carries a high safety risk and requires caution. In addition, Santo André et al. [15] showed that dancers ate fewer calories to maintain a slim figure and body image, and the resulting nutritional imbalance or eating disorders could cause negative physical health experiences, such as dizziness, vomiting, and fainting. This requires attention because it impacts survival, which is vital. In summary, participation in leisure dance activities has been shown to be effective for physical health, but excessive movement and actions to achieve a slim figure could harm participants’ bodies, so caution is needed.

Dancing also affects one’s mental health. Participation in leisure dance activities has a positive effect on mental health. For example, Domene [11] found that participation in Latin dances fostered interest, enjoyment, and a positive psychological outlook, and it enhanced well-being, mood, and health-related quality of life with significant effects. In addition, participation in leisure dance activity enhanced happiness, prosocial attitudes, and self-efficacy [16], improved depressive symptoms [17], reduced stress, and improved cognitive performance and sleep quality [18], leading to positive emotions [19]. Notably, these positive effects of participation in leisure dance activity can improve mental health. However, Jowett et al. [20] confirmed that the pressure to look thin while dancing can lead to feelings of stress, decreased self-confidence, body dissatisfaction, or depression. In summary, participation in leisure dance activity is desirable because it contributes to improving mental health and quality of life for people of all ages, but participants need to enjoy dancing itself, free from the pressure of being thin.

Overall, studies have shown that leisure dance activity has both positive [8,11,12,13,16,17,18,19] and negative effects [14,15,20] on participants, making it important to investigate how leisure dance activity affects the physical and mental health of Koreans participating in leisure dance activity. In this regard, it is necessary to explore the relationship of participation in leisure dance activity with perceived health status (an indicator of physical health) and personal happiness level (an indicator of mental health). Perceived health status refers to how one perceives one’s health status and is used to predict and diagnose one’s physical health [21]. Mansfield et al. [22] conducted a systematic literature review on the association between participation in dance activity and subjective health among 15–24-year-old young people. They found that people’s participation in dance activity positively affected subjective health. Aujla and Needham-Beck [23] found that young dancers with disabilities had no difference in subjective health perceptions before and after participating in a dance program. Meanwhile, happiness has been defined as positive thoughts and feelings about life and is an indicator of one’s mental health [24]. Studies have reported that participation in leisure dance activity is highly associated with improved happiness levels, such as reduced depression symptoms, a greater sense of belonging, and improved social skills among participants [25,26,27,28,29]. However, it was reported that leisure dance activity was not significantly correlated with happiness [30,31], so there were differences between the results of previous studies. Perception of leisure refers to an individual’s subjective idea of leisure and helps in understanding and predicting leisure experiences [32]. The three variables—subjective health, happiness level, and perception of leisure—are important because they are highly relevant to health and impact continued participation in leisure dance activity. Therefore, it is important to explore the relationship between participation in leisure dance activity and these three variables to determine the health effects of individuals’ participation in leisure dance activity and predict their likelihood of continued participation in this activity.

This study examined the associations between participation in leisure dance activities and individual’s perceived health status, happiness, and perceptions of leisure. Specifically, we aimed to explore the effects of leisure dance activity on the physical and mental health of Koreans and to analyze their perceptions of these activities. As a secondary objective, we sought to suggest ways of revitalizing leisure dance activities. It should be noted that these recommendations are not directly derived from empirical data but are based on analytical interpretations. The results of this study are expected to serve as foundational data for the development, improvement, and revitalization of leisure dance activities.

## 2. Materials and Methods

### 2.1. Design and Study Population

This study used data from the 2022 Korea National Leisure Activity Survey organized by the Korean Ministry of Culture, Sports, and Tourism [33]. This survey’s questionnaire, approved by the Korea National Statistical Office [33], has been conducted annually since 2006, and the survey items have been continuously revised and updated. This survey assessed participation in leisure activity, leisure spaces, perceptions of leisure, expenditure on leisure activity, and satisfaction with leisure activity. The 2022 Korea National Leisure Activity Survey was conducted from September to November 2022. It surveyed Koreans aged 15 years or older using stratified sampling. The survey was conducted based on 10,046 Koreans living in 17 cities and provinces across the country.

**Figure 1:**
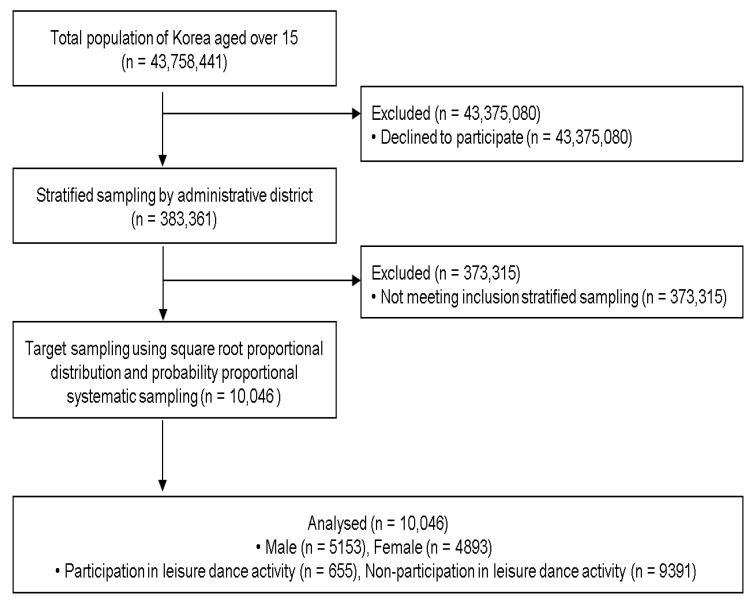
Research flowchart.

The participants were informed about the survey schedule in advance, and interviews were conducted using tablet PCs at the participants’ homes. Random telephone verification was performed on the recovered questionnaires to ensure the accuracy of responses. The questionnaires that passed were subjected to secondary verification by a computerized program, and the questionnaires that were not accepted were supplemented and re-examined. The collected data were entered electronically through the encoding process, and only the data that passed the final test were compiled in the multi-stage verification process. The results of this survey can be accessed from the Korean government’s data-sharing website for academic use. The data do not contain personally identifiable information, such as names, home addresses, phone numbers, or email addresses (https://www.data.go.kr/data/3075652/fileData.do#, accessed on 1 January 2025). Instead, they contain ID numbers. All research procedures were approved by the Korean Ministry of Culture, Sports, and Tourism (approval number: 113014; date: 1 March 2022), and this study was conducted according to the principles outlined in the Declaration of Helsinki. The research flowchart is shown in Figure 1.

### 2.2. Measures

#### 2.2.1. Independent Variables

The independent variable was participation in a leisure dance activity. Participation in leisure dance activities was determined by asking respondents the following question: “Which leisure activity did you participate in at least once in the last year?” There were 88 response options, including dance sports (such as tango, waltz, and jive), dancing (such as ballet, contemporary dance, street dance, b-boying, and broadcast dance), Tae-Bo (including Pilates and yoga), combat sports (such as taekwondo, judo, and kendo), athletics (including jogging and trotting), golf, swimming, hiking, fishing, walking, listening to music, shopping, cooking, and watching television. Those who selected responses under dance sports, dancing, or Tae-Bo were considered to have participated in leisure dance activities, while those who selected other responses were considered not to have participated in leisure dance activities. Tae-Bo is an engaging and highly effective type of aerobic training that combines elements of taekwondo, karate, boxing, ballet, and hip-hop dance with music [34]. Owing to these characteristics, Tae-Bo has been classified as a dance sport.

#### 2.2.2. Dependent Variables

The study included the following dependent variables: demographic characteristics, perceived health status, happiness levels, and perceptions of leisure (positive influence on life, leisure life satisfaction, leisure expense sufficiency, and leisure policy satisfaction). The demographic characteristics variables consisted of gender, age, education level, marital status, residential area, and employment status. Male or female were the categories for gender. Age groups were as follows: 15–19, 20–29, 30–39, 40–49, 50–59, 60–69, or 70 and older. Educational level was classified as elementary school graduate, middle school graduate, high school graduate, or college graduate and above. Marital status was coded as unmarried (1 point); married (2 points); or separated, divorced, or other (3 points). Residential areas were classified as large-, medium-, or small-sized cities. Employed or unemployed were the categories for employment status.

Perceived health status was assessed by asking the respondents the following question: “How do you feel about your health?” Responses were rated on a 7-point Likert scale: very unhealthy (1 point), unhealthy (2 points), slightly unhealthy (3 points), normal (4 points), slightly healthy (5 points), healthy (6 points), and very healthy (7 points). Higher scores indicated more positive perceptions of one’s health status, with responses categorized as follows: very unhealthy, unhealthy, and slightly unhealthy were categorized as “unhealthy” (1 point); normal was categorized as “neutral” (2 points); slightly healthy, healthy, and very healthy were categorized as “healthy” (3 points).

Happiness level was measured by asking the respondents the following question: “How happy are you right now?” Responses were rated on a 10-point Likert scale ranging from unhappy (1 point) to happy (10 points). Higher scores suggested greater happiness levels, with responses categorized as follows: 1–3 points, unhappy (1 point); 4–6 points, neutral (2 points); 7–10 points, happy (3 points).

Perceptions of leisure were assessed using the following variables: positive influence on life, leisure life satisfaction, leisure expense sufficiency, and leisure policy satisfaction. The influence of leisure on life was measured by asking the respondents the following question: “How does leisure activity affect your life?” Responses were rated on a 7-point Likert scale ranging from negative (1 point) to positive (7 points). Higher scores indicated a more positive impact on life, with responses categorized as follows: 1–3 points, negative (1 point); 4 points, neutral (2 points); 5–7 points, positive (3 points). Leisure life satisfaction was measured by asking the respondents the following question: “Are you satisfied with your leisure life?” Responses were rated on a 7-point Likert scale ranging from very dissatisfied (1 point) to very satisfied (7 points). Higher scores indicated higher levels of satisfaction, with responses categorized as follows: 1–3 points, dissatisfied (1 point); 4 points, neutral (2 points); 5–7 points, satisfied (3 points). The variable of leisure expense sufficiency was used to assess whether participants felt that the amount of money they spent on leisure activity was insufficient for their satisfaction or that the money was sufficient because they were satisfied; it was assessed by asking the respondents the following question: “How do you feel about your leisure expenses over the past year?” Responses were rated on a 7-point Likert scale ranging from very insufficient (1 point) to very sufficient (7 points). Higher scores indicated higher levels of sufficiency, with responses categorized as follows: 1–3 points, insufficient (1 point); 4 points, neutral (2 points); 5–7 points, sufficient (3 points). Leisure policy satisfaction, which concerns whether the leisure facilities and related leisure policies are satisfactory, was assessed by asking the respondents the following question: “Are you satisfied with the policies related to leisure facilities in your leisure life?” Responses were rated on a 7-point Likert scale ranging from very dissatisfied (1 point) to very satisfied (7 points). Higher scores indicated higher levels of satisfaction, with responses categorized as follows: 1–3 points, dissatisfied (1 point); 4 points, neutral (2 points); 5–7 points, satisfied (3 points). Leisure policies are developed by government agencies related to leisure with the aim of increasing people’s participation in leisure activities, creating leisure spaces, managing leisure policy processes, and securing leisure environments [32]. The goal of leisure policy is to improve the quality of life of individuals, but it has been noted that Korea’s leisure policy is provider-centered. Therefore, satisfaction with leisure policies is linked to satisfaction and persistence in leisure activities, making it an important topic for this study. The method used in the dependent variables was carried out by utilizing various methods from previous research [35,36].

### 2.3. Statistical Analyses

The collected data were analyzed as follows: First, frequency analyses were conducted to determine the characteristics of the study population. Second, chi-squared tests were performed to identify differences in population characteristics based on participation in leisure dance activities. Third, multivariate logistic regression analyses were conducted to examine the association between participation in leisure dance activities and perceived health status, happiness, perception of leisure, and demographic characteristics. These analyses were performed using SPSS for Windows (version 23.0; IBM Corp., Armonk, NY, USA) to examine the relationships between participation in leisure dance activities and perceived health status, happiness, perception of leisure, and demographic characteristics. In the analysis of the relationships between participation in leisure dance activities and perceived health status, happiness, and perception of leisure, demographic characteristics were used as adjusted variables. All variables were categorized and treated as nominal according to the research purpose. For each dependent variable, a reference category was designated, and odds ratios were calculated to estimate the relative effect of the independent variables on the likelihood of belonging to each category.

Odds ratios (ORs), 95% confidence intervals (CIs), and *p*-values were calculated. In logistic regression analysis, odds and ORs were primarily used to express the trend of dependent variable in relation to an independent variable. Odds refer to the probability that a specific event will occur relative to the probability that it will not occur due to a certain factor. If the probability of the event occurring is p, the odds are calculated as follows:

$$\mathrm{Odds}\;=\frac{P}{1-p}(p=\mathrm{Probability}\mathrm{of}\mathrm{an}\mathrm{incident}\mathrm{occurring},1-p=\mathrm{Probability}\mathrm{of}\mathrm{the}\mathrm{event}\mathrm{not}\mathrm{occurring})$$

OR is a statistical measure used to compare the probability of an event occurring in the presence versus the absence of a specific factor. As its name suggests, it represents the ratio of odds. An OR less than 1 indicates that the factor decreases the likelihood of the dependent variable, whereas an OR greater than 1.000 suggests an increased likelihood. For example, if the OR of men participating in leisure dance activities is 1 and that for women is 13, this indicates that women are 13 times more likely to participate than men. All statistical analyses were performed using SPSS for Windows (version 23.0; IBM Corp., Armonk, NY, USA). Statistical significance was set at the *p* < 0.05 level.

## 3. Results

### 3.1. Characteristics of the Study Population

Table 1 presents the characteristics of the study population. Of the 10,046 individuals, most were woman (51.3%) and did not participate in leisure dance activity (93.5%). Furthermore, most individuals perceived their health status as healthy (69.6%), felt that they were happy (67.6%), believed that leisure has a positive influence on life (88.3%), and were satisfied with their leisure life (56.7%).

### 3.2. Differences in the Characteristics of the Study Population Based on Participation in Leisure Dance Activity

Table 2 presents the results of the chi-squared tests. Gender (χ^2^ = 435.222, *p* < 0.001), age (χ^2^ = 132.635, *p* < 0.001), education level (χ^2^ = 90.342, *p* < 0.001), marital status (χ^2^ = 47.375, *p* < 0.001), residential area (χ^2^ = 20.956, *p* < 0.001), perceived health status (χ^2^ = 36.354, *p* < 0.001), happiness level (χ^2^ = 58.442, *p* < 0.001), positive influence on life (χ^2^ = 17.966, *p* < 0.001), leisure life satisfaction (χ^2^ = 57.867, *p* < 0.001), leisure expense sufficiency (χ^2^ = 13.044, *p* = 0.001), and leisure policy satisfaction (χ^2^ = 17.553, *p* < 0.001) differed significantly based on participation in leisure dance activity. The results of the chi-squared tests imply that the research participants’ participation in leisure dance activity differs according to gender, age, education level, marital status, residential area, perceived health status, happiness level, positive influence on life, leisure life satisfaction, leisure expense sufficiency, and leisure policy satisfaction. Employment status was not statistically significant in the chi-squared tests; therefore, multivariate logistic regression analyses were not included as a variable.

### 3.3. Association Between Participation in Leisure Dance Activity and Demographic Characteristics of the Study Population

**Figure 2:**
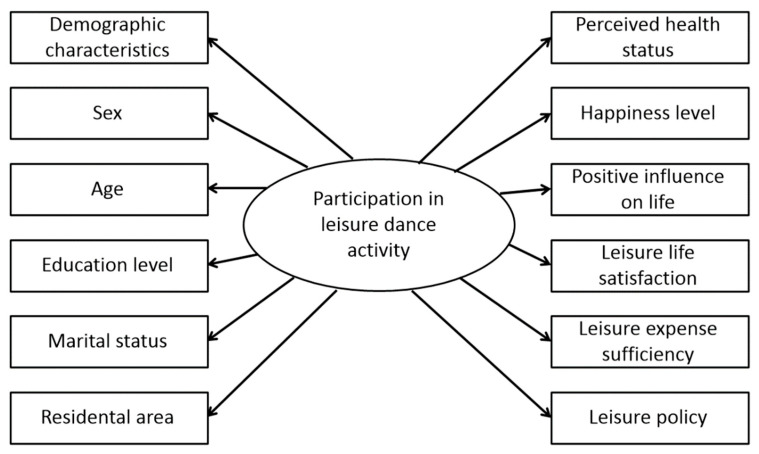
Directed acyclic graph illustrating the relationship between participation in leisure dance activity, perceptions of health and leisure, and demographic characteristics.

**Figure 3:**
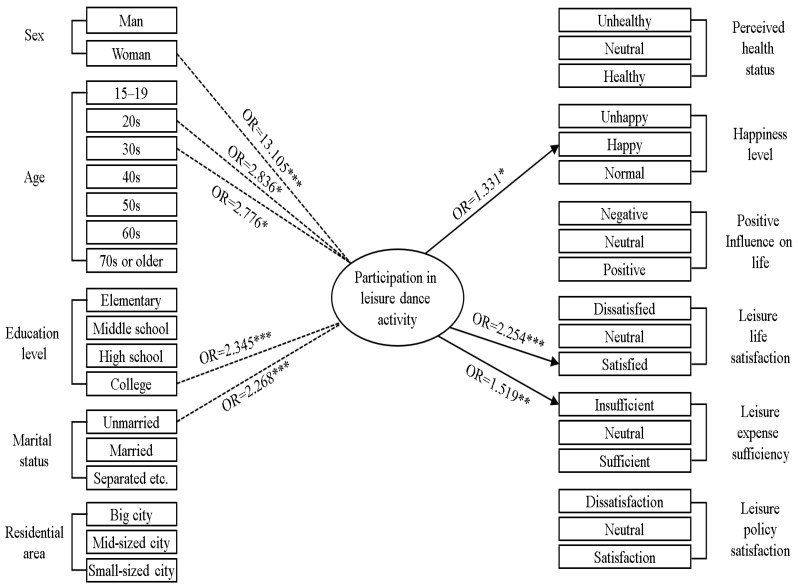
Association among participation in leisure dance activity, demographic characteristics, and health outcomes (* *p* < 0.05; ** *p* < 0.01; *** *p* < 0.001); OR, odds ratio.

Table 3 and Figure 2 present the results of analyzing the association between participation in leisure dance activity and the demographic characteristics of the study population. Figure 3 presents directed acyclic graph illustrating the relationship between participation in leisure dance activity (independent variable), perceptions of health and leisure (dependent variables), and demographic characteristics (adjustment variables).

The OR of being woman was 13.105 (95% CI: 9.993–17.187, *p* < 0.001), indicating that women are more likely to participate in leisure dance activity. The ORs of being in one’s 20s and 30s were 2.836 (95% CI: 1.220–6.590, *p* = 0.015) and 2.776 (95% CI: 1.157–6.659, *p* = 0.022), respectively, suggesting that individuals in their twenties and thirties are more likely to participate in a leisure dance activity. The OR of being a college graduate or having lower educational qualifications was 2.345 (95% CI: 1.568–3.510, *p* < 0.001), indicating that college graduates or those with lower educational achievements are more likely to participate in leisure dance activity. The OR of being unmarried was 2.268 (95% CI: 1.482–3.472, *p* < 0.001), implying that unmarried individuals are more likely to participate in leisure dance activity. No significant association was found between participation in leisure dance activity and residential area (*p* > 0.05).

### 3.4. Association Between Participation in Leisure Dance Activity and Health Outcomes

#### 3.4.1. Effects of Participation in Leisure Dance Activity on Perceived Health Status

Table 4 and Figure 2 present the results of logistic regression analyses examining the effect of participation in leisure dance activity on perceived health status. No significant association was found between participation in leisure dance activity and perceived health status (*p* > 0.05).

#### 3.4.2. Effects of Participation in Leisure Dance Activity on Happiness Levels

Table 5 and Figure 2 present the results of logistic regression analyses examining the effect of participation in leisure dance activity on happiness levels. The OR of feeling that one was happy was 1.331 (95% CI: 1.060–1.672, *p* < 0.001), suggesting that participation in leisure dance activity increases the likelihood of feeling happy.

#### 3.4.3. Effects of Participation in Leisure Dance Activity on Perceptions of Leisure

Table 6 and Figure 2 present the results of logistic regression analyses examining the effect of participation in leisure dance activity on perceptions of leisure and summarizes the results for multiple dependent variables related to leisure perceptions. The OR of feeling satisfied with one’s leisure life was 2.254 (95% CI: 1.550–3.279, *p* < 0.001), indicating that participation in leisure dance activity increases the likelihood of feeling satisfied with one’s leisure life. The OR of perceiving insufficient leisure expenses was 1.519 (1.158–1.993, *p* = 0.003), suggesting that participation in leisure dance activity is more likely to make participants feel that their leisure expenses were insufficient for satisfaction. The participants spent money on leisure dance activities, but they felt the satisfaction they received was not sufficient because of financial constraints. No significant association was found between participation in leisure dance activity and positive influence on life, and between participation in leisure dance activity and leisure policy satisfaction (*p* > 0.05).

## 4. Discussion

### 4.1. Interpretation of the Findings

#### 4.1.1. The Conditions of Leisure Dancing Activity

Our results showed that women are more likely to participate in leisure dance activity. This aligns with the broader perspective that dance, including ballet and contemporary dance, is a feminine activity [37]. Dancing is generally considered a socially acceptable activity for girls but not for boys. From an early age, young girls are encouraged to pursue dancing, whereas boys tend to avoid “anything that is feminine, homosexual, or unmasculine” [38]. These gendered social norms surrounding dance are likely to lead to greater participation by women than men. Similarly, our finding shows that, due to the incorrect perceptions of dancing learned from an early age, leisure dance activity has become a women-exclusive activity, and there is an invisible wall for men. Men who enjoy leisure dance activity often experience difficulties in learning dance because of these social perceptions and prejudices [39,40]. Consistent with our findings, previous studies have shown gender-based differences in participation in leisure activity. Auhuber et al. [41] found that boys are more physically active than girls in several physical leisure activities, whereas girls are more active in dancing. This is particularly true for some dance forms. With its roots in Middle Eastern and North African countries, belly dancing is a popular leisure activity in the West, and women of all ages and body types engage in it [42]. Overall, women were more likely to participate in leisure dance activity than men. Therefore, it is necessary to accurately identify the factors limiting participation and find ways to facilitate men’s participation in leisure dance activity.

Second, our findings showed that people in their twenties and thirties are more likely to participate in leisure dance activities. To engage in dancing, one must learn the right body movements. This often requires one to enroll in high-cost dance, cultural, or sports centers, making participation in leisure dance activity a costly affair [30,43]. In Korea, individuals in their twenties and thirties are called Generation MZ. Accounting for 45% of the economically active population, Generation MZ tends to participate in leisure activities for personal enjoyment, even if they are costly [44]. Moreover, this generation comprises active consumers who exhibit a high achievement desire attitude, investing time, money, and mental resources to improve their knowledge and skills through leisure [45]. People in their twenties and thirties who participate in leisure dance activities often pay high fees to take one-on-one lessons with an instructor or attend special lectures by professional dancers to improve their dance skills [40]. Although these individuals feel burdened by the costs of attending expensive dance lessons or special lectures, it can be inferred that they are willing to spend money on the activity they want to do because of their penchant for spending. In other words, although it is expensive to participate in leisure dance activities, the tendency of 20–29- and 30–39-year-olds to spare no expense for the activity they want seems to facilitate their participation in leisure dance activity. Leisure dance activity is mostly popular among people in their twenties and thirties, and, considering their age and continuous participation, the age range of those engaging in this activity is expected to widen. However, the participation rate of the older individuals is currently the lowest. Support is needed at the national level to make leisure dance activity accessible to people of various ages, including older individuals.

Third, our results showed that college graduates or individuals with lower educational achievements are more likely to participate in leisure dance activity. Lee and Jo [46] found that the higher the education level, the more the dance-learning experience. More specifically, they found the most dance-learning experience among college graduates, followed by high-school graduates and middle-school graduates, partially supporting the results of this study. Song and Lee [47] found that people who enjoy dancing as a leisure activity choose dancing for reasons such as participating in dance clubs or performing with their friends. It has also been found that those who have had dance-related experiences in elementary school, middle school, high school, or university and those who have extensive knowledge of the dance world are more likely to practice dancing in their adulthood [48,49,50,51]. This is because the movements involved in the dancing process become ingrained in the body and mind [52]. On the other hand, the relatively low likelihood that individuals with low educational qualifications are less likely to participate in leisure dance activity can be linked to the lack of dance activity experience during school. School physical education is the main means of delivering physical activity to students, and many students conduct most of their physical activity through school sports [53]. People who have more experience in physical activity in school sports are more likely to practice physical activity in adulthood [54]. Therefore, individuals with low educational attainment are more likely to lack dance-related experience through school sports and are more likely not to participate in related physical activities as adults. In other words, higher education levels translate to more frequent dance-learning experiences, which ingrain dance movements in one’s body and mind and encourage one to continue dancing as a leisure activity in adulthood. This finding also highlights the necessity and importance of providing dance learning experiences for school-age students.

Fourth, our findings showed that unmarried individuals are more likely to participate in leisure dance activities. Studies have shown that unmarried people spend significantly more time engaging in leisure activity than married people [55,56]. This is because, compared to married people, unmarried people have fewer family-related obligations, such as housework and family care, and are more likely to engage in leisure activities [57]. Moreover, unmarried employed individuals can be expected to participate in leisure dance activities because they possess the free time and economic power necessary for engaging in such activities [58]. In other words, participation in leisure activities differs depending on marital status, and being married may be a limiting factor. Meanwhile, positive effects such as emotional connection and conflict resolution have been reported when couples participate in leisure dance activity together [59]. Especially, it is necessary to guide many couples to know the effect of their participation in leisure dance activity so that they can participate.

#### 4.1.2. The Effects of Leisure Dancing Activity

Our results showed that participation in leisure dance activities is likely to increase happiness levels. Han and Choi [45] qualitatively studied the health and happiness of older women who participated in leisure dance activity. They found that regular participation in this activity not only helps maintain people’s health but also improves their self-image by alleviating depression and poor physical condition and making them feel happy [25]. Lee et al. [26] found a positive relationship between participation in traditional Korean dance and happiness among adult women. The results of this study suggest that engagement in leisure activity is a critical variable in increasing well-being and happiness [26]. Zitomer [27] reported that children with disabilities who received dance education in elementary school experienced a sense of belonging and success when they danced with their peers, which increased their sense of well-being. It has also been reported that older women enjoy line dancing as a leisure activity, and it helps them make new friends and increase their happiness as they feel a sense of accomplishment and confidence [28]. Kuwahara et al. [29] found that participation in physical leisure activity, such as radio gymnastics and aerobics, is highly correlated with reducing depressive symptoms and that consistent participation in physical activity prevents depression. Wei et al. [31] found a positive correlation between leisure time and happiness levels in China. However, they also reported that happiness levels differ based on the type of leisure activity; passive leisure activity (such as watching television and surfing the Internet) contributed to happiness, but active leisure activity (such as exercising, socializing, and shopping) did not [30]. Overall, participation in leisure dance activity provides an opportunity to enhance physical and psychological well-being, and those who participate feel happier than those who do not. However, there seem to be some differences depending on the situation and conditions of the country. Based on the results of this study alone, it can be deduced that the relationship between Korean people’s participation in leisure dance activity and happiness has a significant impact on their mental health.

Second, our results showed that participation in leisure dance activity is more likely to increase leisure life satisfaction. A high level of leisure life satisfaction means that people who participate in leisure dance activity are more likely to continue to participate in the future [60]. Choi and Lee [61] analyzed the relationship between dance sports participation and leisure satisfaction among 172 older adults who participated in dance sports. They found that leisure satisfaction was high in psychological, educational, physiological, and environmental aspects. Lim [62] analyzed the leisure satisfaction of 285 people aged 20–50 who participated in jazz dance. They found that leisure satisfaction was highest among those who participated in jazz dance every day, were aged 30–39, were married, and had a household income of less than USD 2000–3000. Pines and Giles [63] found that adults aged 20–80 who learned ballet had a high level of leisure satisfaction and considered ballet a social activity that helped them feel a sense of community or friendship with others. Additionally, they wanted to continue practicing ballet for a long time because it was a part of their identity. Consistent with the results of this study, people who participated in leisure dance activities, such as dancesport, jazz dance, and ballet, had higher levels of leisure satisfaction. Leisure satisfaction is a major factor that drives continued participation in leisure activity. Therefore, it can be inferred that individuals who participate in leisure dance activities are more likely to continue participating in the future because of high leisure satisfaction [60], and that this activity may encourage participation in leisure activity.

Third, our results showed that leisure dance activity is more likely to be insufficient in terms of expense, where participants spend a lot of money on leisure activities but feel that the expense is too high for the satisfaction obtained. Engaging in leisure activities involves temporal, economic, physical, psychological, and social costs [64]. The more actively one participates in leisure activities, the greater the costs involved. In particular, those who participate in leisure dance activity experience difficulties due to increasing expenses over time; simultaneously, dance becomes a part of their daily routine [40,43,45,65]. For example, people who enjoy ballet as a leisure activity are aware of ballet beyond its original context. However, they spend a substantial amount of money on ballet and integrate it into their lives, such as listening to ballet music, watching ballet performances, and purchasing ballet clothes [65]. This indicates that ballet has become an integral part of their lives. Their leisure activity led them to engage in new activities, such as performing expensive ballets, going to the movies, and buying ballet clothes, which further increases their leisure expenses. As a result, participants may feel that their leisure expenses are insufficient due to the high costs associated with this activity, which are disproportionate to the amount of money they have. In other words, this consumption activity, triggered by a leisure dance activity, is likely to increase the money one spends on leisure activity.

### 4.2. Ways to Revitalize Leisure Dance Activity

Based on the results of this study, we present the following ways through which leisure dance activity can be revitalized. First, high-quality dance education should be provided in schools because dance-related experiences in childhood increase the likelihood of dancing in adulthood [41,49,51]. Of the 10,046 individuals in this study, only 655 participated in leisure dance activity, of whom 3.1% were adolescents. These figures starkly differ from those in other countries. In Australia, dance has the highest participation rate among girls and the second-highest participation rate among boys and girls among all leisure activities [66]. Therefore, Korean youth must be encouraged to participate in leisure dance activities. The low participation of adolescents in leisure dance activity could stem from the Korean education system, in which dance education is a part of physical education, and physical education teachers must also teach dancing. However, often, they lack the professionalism required to provide dance education [67]. As a result, they tend to fail to provide proper dance classes or teach some other sport [67]. Therefore, it has been reported that students feel that there are high barriers to learning dance as a leisure activity, and this inhibits them from engaging in dance in adulthood [68].

Being one of the most expressive art forms, dancing provides aesthetic experiences through body movements. Therefore, dance should be taught more widely in schools so that young people can have aesthetic experiences that improve their quality of life [69]. Considering that school age is particularly important in laying the foundation for personality development, it is necessary to include dance in the curriculum and teach it systematically [70]. These efforts will increase the likelihood of young people enjoying dance as a leisure activity and continuing it in adulthood. This makes it necessary to increase the professionalism of physical education teachers and support them in providing dance education. In other words, because education quality depends on how teachers design and manage their lessons, it is necessary to improve the quality of teachers.

The professionalism of physical education teachers can be improved by providing training programs, distributing casebooks, organizing classes with dance instructors, and creating teacher learning communities. Teacher learning communities, in particular, can not only improve the quality of dance education but also aid teachers’ professional development. Teachers can design and run a dance curriculum together or with a professional dancer to enhance their expertise [71,72]. If physical education teachers design dance classes that take into account students’ development, interests, and motivations, it can attract high participation and improve the quality of dance education [71,72]. Kwon et al. [72] designed and implemented a Korean dance class with a collaborative instructional design to cultivate aesthetic and emotional competence. Consequently, teachers were able to develop their expertise, and students were able to learn the value of movement. In addition, to increase the professionalism of physical education teachers, it is also important for them to develop their expertise even before they become teachers. Teacher training institutions in Korea, which currently train physical education teachers, need to improve the curriculum for dance education. For instance, there are many teacher training institutions where dance classes are elective rather than compulsory, and pre-service physical education teachers who are men sometimes graduate without choosing these classes [73]. In addition, graduation exams require women to take dance and men to take ball sports, separating the subjects they take according to gender, which can make it difficult for physical education teachers who are men to develop their expertise in administering dance classes [73]. Considering that most physical education teachers in Korea are men, the lack of experience in participating in dance classes in the pre-service teacher course is likely to lead to difficulties in managing dance classes. Therefore, there is a need to improve the curriculum so that pre-service teachers can participate in dance classes. Hence, physical education teachers should design dance classes with their colleagues by taking into account students’ development, interests, and motivation. This will lead to meaningful dance experiences among adolescents and create positive perceptions of dancing. It will also make dancing a standard form of leisure activity in Korean society. Second, efforts are required to improve men’s awareness of leisure dance activity and lower barriers to entry. Among the 655 individuals who participated in leisure dance activity in this study, only 9.3% were men. Considering that there are numerous types of leisure dance activities available, only a small percentage of people participate in these activities. Therefore, it is necessary to raise men’s awareness and encourage them to participate in dance activities.

One way of encouraging men’s participation in dance is to combine dance with other activities. The idea is to add dance to an activity that one is used to, so that one can participate in dancing without the fear of societal rejection. More specifically, dance programs can be created in a game format, like Wii Fit, Dance Central, Dance Dance Revolution, and Just Dance. Men can watch the movements, attempt to imitate them, and become familiar with the movements [74]. Dance-based gaming activities have been reported to improve dance skills, which require full-body movements, motor coordination, and aerobic endurance, thus helping people to acquire and develop movement skills [75]. Older adults who participated in Dance ExerGames have also reported that dance improved their physical, cognitive, and psychological well-being [76]. Games that are played with wearable devices allow one to engage in dancing across time and space [77]. This combination of Dance ExerGames and wearable devices is expected to be particularly effective for men, as it would allow them to enjoy dancing at home, avoiding potential judgment from others. Therefore, combining dancing and technology can reduce the barriers men face in participating in leisure dance activity.

### 4.3. Limitations

The limitations of this study are as follows: First, because this study used secondary data, it could not determine temporal antecedents or causal relationships. And the cross-sectional design and the single-time-point survey limits its ability to infer causality. Second, data on perceived health status, happiness level, and perceptions of leisure were self-reported. Participants may overestimate their physical activity or perceived health status. Additionally, their responses may have been influenced by their health conditions. Third, this study used data from a study conducted by the Korean Ministry of Culture, Sports, and Tourism. The validity, reliability, and quality of the questionnaires are limited by the fact that they were not verified internally. The absence of established scales or reliability indices further limits the interpretive value of the findings. Despite the large overall sample size, the very low proportion of leisure dance participants leads to highly unbalanced groups, which could bias regression estimates and reduce statistical power. However, since the questionnaire used in this study was administered by a government agency, it is undergoing a process of periodic revision and improvement by experts, and various studies have been conducted using this questionnaire. Fourth, only 6.5% of the participants in this study participated in leisure dance activities, limiting the generalizability of the study’s findings. Although the study targeted only 6.5% of those who participated in leisure dance activities, the number of participants was 10,046, which is significant in that the study was conducted based on a large number of participants. Fifth, the degree of participation (e.g., frequency and/or intensity) can have different impacts on physical, social, and psychological parameters. The independent variable is treated as binary, without consideration of key factors such as frequency, duration, or intensity, which are essential to understanding potential associations with health and well-being. Moreover, the dependent variables rely on single-item measures from a national survey. To improve generalizability, additional analyses such as Cramer’s V, Nagelkerke’s R-squared, power estimation based on sample size, and minimal sample size calculations should be performed. However, since the independent variable of this study is participation in leisure dance activities, it does not affect the results. Sixth, the data presentation is primarily a simple comparison between participants who engage in leisure dance activities and those who do not, without conducting any analyses examining the relationships among the various variables. This approach may not fully align with the established guidelines for multivariate data analysis, which could lead to a misrepresentation of the underlying data. A more robust examination of the relationships between these variables would enhance the validity of our findings within the context of this dataset. Seventh, concept of ‘Leisure dance activity’ is treated as a broad category, without distinguishing between types (e.g., ballroom, K-pop, traditional, freestyle). However, the results of this study cannot be generalized. The operationalization of leisure dance activities is overly broad. This includes activities such as Tae-Bo, Pilates, and yoga, which are not strictly dance forms. This aggregation may blur conceptual boundaries and reduce construct validity. Finally, the associated *p*-value, while statistically significant due to the large sample size, suggests an effect size that may lack practical significance. Despite these limitations, this study is significant because it investigated the relationship between participation in leisure dance activity and perceived health status, happiness level, and perceptions of leisure based on actual data of Koreans’ participation in leisure activities and explored ways to revitalize leisure dance activities. In the future, the following studies should be conducted: First, research should focus on qualitative studies that explore, in depth, the changes in participants’ health and lives before and after engaging in leisure dance activities. Second, studies should investigate the effects of participating in leisure dance activity using wearable devices and exergames on perceived health status, happiness levels, and perceptions of leisure. Third, there is a need for research to specifically explore the relationship between frequency or intensity of participation and variables such as the number of days and duration of leisure dance activity.

## 5. Conclusions

The purpose of this study was to examine the associations between participation in leisure dance activity and perceived health status, happiness levels, and perceptions of leisure, as well as explore ways of revitalizing leisure dance activity. Data were collected from the 2022 Korea National Leisure Activity Survey and analyzed using frequency analysis, chi-squared tests, and multivariate logistic regression analysis. In this study, we found no significant association between participation in leisure dance activity and perceived health status. However, participation in leisure dance activity was more likely to make individuals feel happy, satisfied with their leisure life, and feel that their leisure expenses are insufficient. To encourage students’ participation in leisure dance activity, schools should enhance physical education teachers’ expertise in providing high-quality dance classes. Additionally, efforts should be made to increase men’s awareness and perceptions of leisure dance activity and lower barriers to entry. This can be achieved by combining dance, games, and technology.

## Figures and Tables

**Table 1:** Characteristics of the study population (n = 10,046).

Characteristic	Categories	n (%)
Gender	Man	4893 (48.7%)
	Woman	5153 (51.3%)
Age	15–19	487 (4.8%)
	20–29	1371 (13.6%)
	30–39	1433 (14.3%)
	40–49	1717 (17.1%)
	50–59	1831 (18.2%)
	60–69	1707 (17.1%)
	70 or older	1500 (14.9%)
Education level	Elementary school graduate	1146 (11.4%)
	Middle school graduate	1136 (11.3%)
	High school graduate	3790 (37.7%)
	College graduate or less	3974 (39.6%)
Marital status	Unmarried	2704 (26.9%)
	Married	6093 (60.7%)
	Separated, divorced, or other	1249 (12.4%)
Residential area	Big city	4331 (43.1%)
	Mid-sized city	3459 (34.4%)
	Small-sized city	2256 (22.5%)
Employment status	Employed	5657 (56.3%)
	Unemployed	4389 (43.7%)
Participation in leisure dance activity	Yes	655 (6.5%)
	No	9391 (93.5%)
Perceived health status	Unhealthy	764 (7.6%)
	Neutral	2288 (22.8%)
	Healthy	6994 (69.6%)
Happiness level	Unhappy	106 (1.1%)
	Neutral	3152 (31.3%)
	Happy	6788 (67.6%)
Positive influence on life	Negative	96 (1.0%)
	Neutral	1077 (10.7%)
	Positive	8873 (88.3%)
Leisure life satisfaction	Dissatisfied	1048 (10.5%)
	Neutral	3300 (32.8%)
	Satisfied	5698 (56.7%)
Leisure expense sufficiency	Insufficient	1043 (10.4%)
	Neutral	3669 (36.5%)
	Sufficient	5334 (53.1%)
Leisure policy satisfaction	Dissatisfied	878 (8.7%)
	Neutral	3903 (38.9%)
	Satisfied	5265 (52.4%)

**Table 2:** Difference in the characteristics of the study population based on participation in leisure dance activity (n = 10,046).

Characteristic	Categories	Participation in Leisure Dance Activity	Non-Participation in Leisure Dance Activity	χ^2^ (*p*)
Gender	Man	61 (9.3%)	4832 (51.5%)	435.222 (<0.001 ***)
	Woman	594 (90.7%)	4559 (48.5%)	
Age	15–19	20 (3.1%)	467 (5.0%)	132.635 (<0.001 ***)
	20–29	159 (24.3%)	1212 (12.9%)	
	30–39	142 (21.7%)	1291 (13.7%)	
	40–49	109 (16.6%)	1608 (17.1%)	
	50–59	103 (15.7%)	1728 (18.4%)	
	60–69	74 (11.3%)	1633 (17.4%)	
	70 or older	48 (7.3%)	1452 (15.5%)	
Education level	Elementary school graduate	37 (5.6%)	1109 (11.8%)	90.342 (<0.001 ***)
	Middle school graduate	39 (6.0%)	1097 (11.7%)	
	High school graduate	213 (32.5%)	3577 (38.1%)	
	College graduate or less	366 (55.9%)	3608 (38.4%)	
Marital status	Unmarried	345 (37.4%)	2459 (26.2%)	47.375 (<0.001 ***)
	Married	363 (55.4%)	5730 (61.0%)	
	Separated, divorced, or other	47 (7.2%)	1202 (12.8%)	
Residential area	Big city	319 (48.7%)	4012 (42.7%)	20.956 (<0.001 ***)
	Mid-sized city	235 (35.9%)	3224 (34.4%)	
	Small-sized city	101 (15.4%)	2155 (22.9%)	
Employment status	Employed	349 (53.3%)	5308 (56.5%)	2.612 (0.058)
	Unemployed	306 (46.7%)	4083 (43.5%)	
Perceived health status	Unhealthy	28 (4.3%)	736 (7.8%)	36.354 (<0.001 ***)
	Neutral	103 (15.7%)	2185 (23.3%)	
	Healthy	524 (80.0%)	6470 (68.9%)	
Happiness level	Unhappy	3 (0.5%)	103 (1.1%)	58.442 (<0.001 ***)
	Neutral	121 (18.5%)	3031 (32.3%)	
	Happy	531 (81.1%)	6257 (66.6%)	
Positive influence on life	Negative	1 (0.2%)	95 (1.0%)	17.966 (<0.001 ***)
	Neutral	43 (6.6%)	1034 (11.0%)	
	Positive	611 (93.2%)	8262 (88.0%)	
Leisure life satisfaction	Dissatisfied	36 (5.5%)	1012 (10.8%)	57.867 (<0.001 ***)
	Neutral	156 (23.8%)	3144 (33.5%)	
	Satisfied	463 (70.7%)	5235 (55.7%)	
Leisure expense sufficiency	Insufficient	80 (12.2%)	963 (10.2%)	13.044 (0.001 **)
	Neutral	197 (30.1%)	3472 (37.0%)	
	Sufficient	378 (57.7%)	4956 (52.8%)	
Leisure policy satisfaction	Dissatisfied	49 (7.5%)	829 (8.8%)	17.553 (<0.001 ***)
	Neutral	211 (32.2%)	3692 (39.3%)	
	Satisfied	395 (60.3%)	4870 (51.9%)	

** *p* < 0.01, *** *p* < 0.001; tested using chi-squared tests.

**Table 3:** Logistic regression results for demographic characteristics.

Characteristic	Categories	Odds Ratio (95% Confidence Intervals)	*p*-Value
Gender	Man	1.000	
	Woman	13.105 (9.993–17.187)	<0.001 ***
Age	15–19	1.000	
	20–29	2.836 (1.220–6.590)	0.015 *
	30–39	2.776 (1.157–6.659)	0.022 *
	40–49	1.956 (0.804–4.760)	0.139
	50–59	2.187 (0.894–5.351)	0.086
	60–69	2.110 (0.849–5.246)	0.108
	70 or older	2.207 (0.838–5.815)	0.109
Education level	Elementary school graduate	1.000	
	Middle school graduate	0.873 (0.535–1.423)	0.585
	High school graduate	1.346 (0.901–2.010)	0.147
	College graduate or less	2.345 (1.568–3.510)	<0.001 ***
Marital status	Unmarried	2.268 (1.482–3.472)	<0.001 ***
	Married	1.325 (0.934–1.880)	0.114
	Separated, divorced, or other	1.000	
Residential area	Big cities	1.204 (0.975–1.532)	0.132
	Mid-sized city	1.152 (0.897–1.480)	0.267
	Small-sized city	1.000	

* *p* < 0.05, *** *p* < 0.001; tested using multivariate logistic regression analysis adjusted for gender, age, education level, marital status, residential area, employment status.

**Table 4:** Logistic regression results for perceived health status.

Variable	Perceived Health Status	Odds Ratio (95% Confidence Intervals)	*p*-Value
Dance participation (yes)	Unhealthy	1.054 (0.675–1.646)	0.817
	Neutral	0.830 (0.655–1.052)	0.122
	Healthy	1.000	

Tested using multivariate logistic regression analysis adjusted for gender, age, education level, marital status, residential area, employment status.

**Table 5:** Logistic regression results for happiness level.

Variable	Happiness Level	Odds Ratio (95% Confidence Intervals)	*p*-Value
Dance participation (yes)	Unhappy	1.101 (0.328–3.689)	0.876
	Happy	1.331 (1.060–1.672)	0.014 *
	Normal	1.000	

* *p* < 0.05; tested using multivariate logistic regression analysis adjusted for gender, age, education level, marital status, residential area, employment status.

**Table 6:** Logistic regression results for perceptions of leisure.

Variable	Dependent Variable	Categories	Odds Ratio (95% Confidence Intervals)	*p*-Value
Dance participation (yes)	Positive influence on life	Negative	0.228 (0.031–1.670)	0.146
		Neutral	0.886 (0.636–1.241)	0.487
		Positive	1.000	
	Leisure life satisfaction	Dissatisfied	1.000	
		Neutral	1.401 (0.956–2.053)	0.084
		Satisfied	2.254 (1.550–3.279)	<0.001 ***
	Leisure expense sufficiency	Insufficient	1.519 (1.158–1.993)	0.003 **
		Neutral	0.879 (0.728–1.060)	0.177
		Sufficient	1.000	
	Leisure policy satisfaction	Dissatisfied	0.871 (0.632–1.201)	0.400
		Neutral	0.834 (0.695–1.001)	0.052
		Satisfied	1.000	

** *p* < 0.01, *** *p* < 0.001; tested using multivariate logistic regression analysis adjusted for gender, age, education level, marital status, residential area, employment status.

## Data Availability

Publicly available datasets were analyzed in this study. This data can be found here: [https://www.data.go.kr/data/3075652/fileData.do#], accessed on 1 January 2025.
